# Adjuvants and Immunization Strategies to Induce Influenza Virus Hemagglutinin Stalk Antibodies

**DOI:** 10.1371/journal.pone.0079194

**Published:** 2013-11-06

**Authors:** Peter H. Goff, Dirk Eggink, Christopher W. Seibert, Rong Hai, Luis Martínez-Gil, Florian Krammer, Peter Palese

**Affiliations:** 1 Department of Microbiology, Icahn School of Medicine at Mount Sinai, New York, New York, United States of America; 2 Graduate School of Biomedical Sciences, Icahn School of Medicine at Mount Sinai, New York, New York, United States of America; 3 Department of Medicine, Icahn School of Medicine at Mount Sinai, New York, New York, United States of America; Shanghai Medical College, Fudan University, China

## Abstract

The global population remains vulnerable in the face of the next pandemic influenza virus outbreak, and reformulated vaccinations are administered annually to manage seasonal epidemics. Therefore, development of a new generation of vaccines is needed to generate broad and persistent immunity to influenza viruses. Here, we describe three adjuvants that enhance the induction of stalk-directed antibodies against heterologous and heterosubtypic influenza viruses when administered with chimeric HA proteins. Addavax, an MF59-like nanoemulsion, poly(I:C), and an RNA hairpin derived from Sendai virus (SeV) Cantell were efficacious intramuscularly. The SeV RNA and poly(I:C) also proved to be effective respiratory mucosal adjuvants. Although the quantity and quality of antibodies induced by the adjuvants varied, immunized mice demonstrated comparable levels of protection against challenge with influenza A viruses on the basis of HA stalk reactivity. Finally, we present that intranasally, but not intramuscularly, administered chimeric HA proteins induce mucosal IgA antibodies directed at the HA stalk.

## Introduction

Influenza viruses cause substantial annual morbidity and mortality with seasonal epidemic outbreaks of influenza A subtypes H1 and H3 and influenza B viruses as the etiologic agents in the vast majority of human cases. Influenza A viruses also periodically cause global pandemics which have occurred 4 times in the past century including the 1918 Spanish influenza (H1N1), 1957 Asian influenza (H2N2), 1968 Hong Kong (H3N2) influenza and most recently, the 2009 swine origin influenza pandemic (pH1N1) [1]. Seasonal influenza epidemics may be managed by vaccination, and trivalent vaccines containing H1N1 and H3N2 influenza A components plus an influenza B component have been most widely distributed [2]. However, this immunization strategy relies upon accurate prediction of the next seasonal viruses to circulate in order to reformulate and manufacture the vaccine each year. Accurate prediction is challenging and mismatches are common [3]. Furthermore, trivalent vaccines may be of limited efficacy even in well matched years, and they do not protect against strains that have undergone significant drift, heterosubtypic strains or potential pandemic viruses [4]. The correlate of protective immunity for traditional influenza vaccines is a hemagglutination inhibiting (HAI) humoral response to the immunodominant globular head of influenza hemagglutinin [5]. While the majority of neutralizing antibodies target epitopes in the globular head domain, its antigenic regions are highly variable and continually escape the human immune system’s humoral response [2]. 

 Therefore, the highly conserved, but immunosubdominant, stalk region of HA is an attractive target for universal vaccine development. Many stalk epitopes are conserved throughout group I influenza viruses as evidenced by the broadly cross-reactive monoclonal antibodies that have been published in the last several years [6]. Our lab has developed and described chimeric influenza hemagglutinins (cHA) which consist of a globular head displayed on the stalk of another subtype; for example, an H5 head on an H1 stalk is referred to as cH5/1 [7]. The use of the chimeric constructs allows for proper folding and stabilization of conserved epitopes present in functional HA trimers [7], and they also represent a powerful tool for detecting HA stalk-specific antibodies [8]. A sequential vaccination strategy with different cHAs was designed for repeated exposure to a single stalk while utilizing a unique globular head for each immunization to limit the immune response toward the immunodominant globular head. We have previously demonstrated that such a vaccine strategy is protective against influenza virus challenge in mice [9].

The most widely distributed influenza vaccines in the United States are currently unadjuvanted, although effective adjuvants are a means of inducing broader seroreactivity to HA subunit vaccines [10,11]. However, the use of adjuvants for boosting HA stalk-specific antibodies warrants further exploration. Herein, we characterize the use of soluble, trimerized HA protein administered either intramuscularly (IM) or intranasally (IN) with a variety of adjuvants and define a broad range of induced HA stalk seroreactivity. We have previously described the use of an *in vitro* transcribed (IVT) RNA hairpin derived from the defective interfering (DI) RNA of the Sendai virus (SeV) Cantell strain as an effective influenza virus vaccine adjuvant [12]. The adjuvant effectively stimulates humoral immunity and protects mice against challenge with a virus homologous to the vaccine on the basis of reactivity HA globular head. We sought to determine whether IVT SeV DI RNA, Addavax, an MF59-like nanoemulsion, and poly(I:C) can effectively boost stalk-directed immunity and induce a broadly reactive seroresponse in combination with soluble cHA protein. By exploring stalk-directed vaccine strategies in combination with different adjuvants, we demonstrate that adjuvants play a critical role in the development of a cross protective humoral response to the HA stalk domain.

## Methods

### Ethics

All mouse experiments were approved by and performed under the guidelines of the Icahn School of Medicine at Mount Sinai Institutional Animal Care and Use Committee (permit # LA09-00266). Appropriate care was taken to ensure the animals’ welfare and humane endpoints. 

### Cells and viruses

 293T and MDCK cells (ATCC) were cultured in Dulbecco’s Modified Eagle medium (DMEM, Gibco) with 10% fetal calf serum (HyClone), 100 units/mL penicillin and 100 µg/mL of streptomycin (Pen/Strep, Gibco). Recombinant cH5/1 and cH9/1 viruses were produced via reverse genetics in the PR8 background as ‘7+1’ reassortants [7]. A ‘6+2’ recombinant virus expressing a low pathogenicity HA with the polybasic cleavage site removed and NA from influenza A/Vietnam/1203/2004 (H5N1) was also rescued in the PR8 background. These viruses and other isolates utilized, including influenza A/Netherlands/602/2009 (pH1N1, mouse adapted), A/Puerto Rico/8/1934 (PR8), A/USSR/90/1977 and A/Brisbane/59/2007, were propagated in 8 - 10 day old embryonated eggs at 37 °C for 48 hours under BSL2 conditions. A ‘6+2’ recombinant H2N2 virus with the HA and NA from A/Singapore/1/1957 in the cold adapted Ann Arbor (A/Ann Arbor/6/60) background was grown in 8 day old embryonated eggs at 33 °C for 48 hours and maintained under BSL2+ conditions in accordance with Mount Sinai’s institutional guidelines. All viruses were titered on MDCK cells using the plaque assay.

### Animals

 Female BALB/c mice, aged 6-8 weeks at the initiation of each vaccine regimen, were housed on a 12 hour light-dark cycle with uninterrupted access to food and water. Mice were anesthetized with 0.1 mL of ketamine/xylazine administered intraperitoneally for intranasal vaccination or viral infection.

### Chimeric hemagglutinin proteins

Soluble, trimerized cH5/1, cH6/1, cH9/1 and H1 proteins were generated with a baculovirus expression system and purified as previously described [7,13]. The globular head for cH6/1 is derived from A/mallard/Sweden/81/02 and the cH9/1 globular head is from A/guinea fowl/Hong Kong/WF10/99. Each of these cHAs utilize the PR8 stalk, and the junctions between the globular head and stalk domains used for cloning the recombinant cHAs are amino acid residues C52 and C277 (PR8 numbering). Primer sequences utilized for cloning the cHAs are available upon request. Full length PR8 HA was used for the H1 protein. Proteins are expressed as trimers in order to maintain native, conformational epitopes as confirmed by monoclonal antibody 6F12 binding [14].

### Vaccination

**Figure 1:**
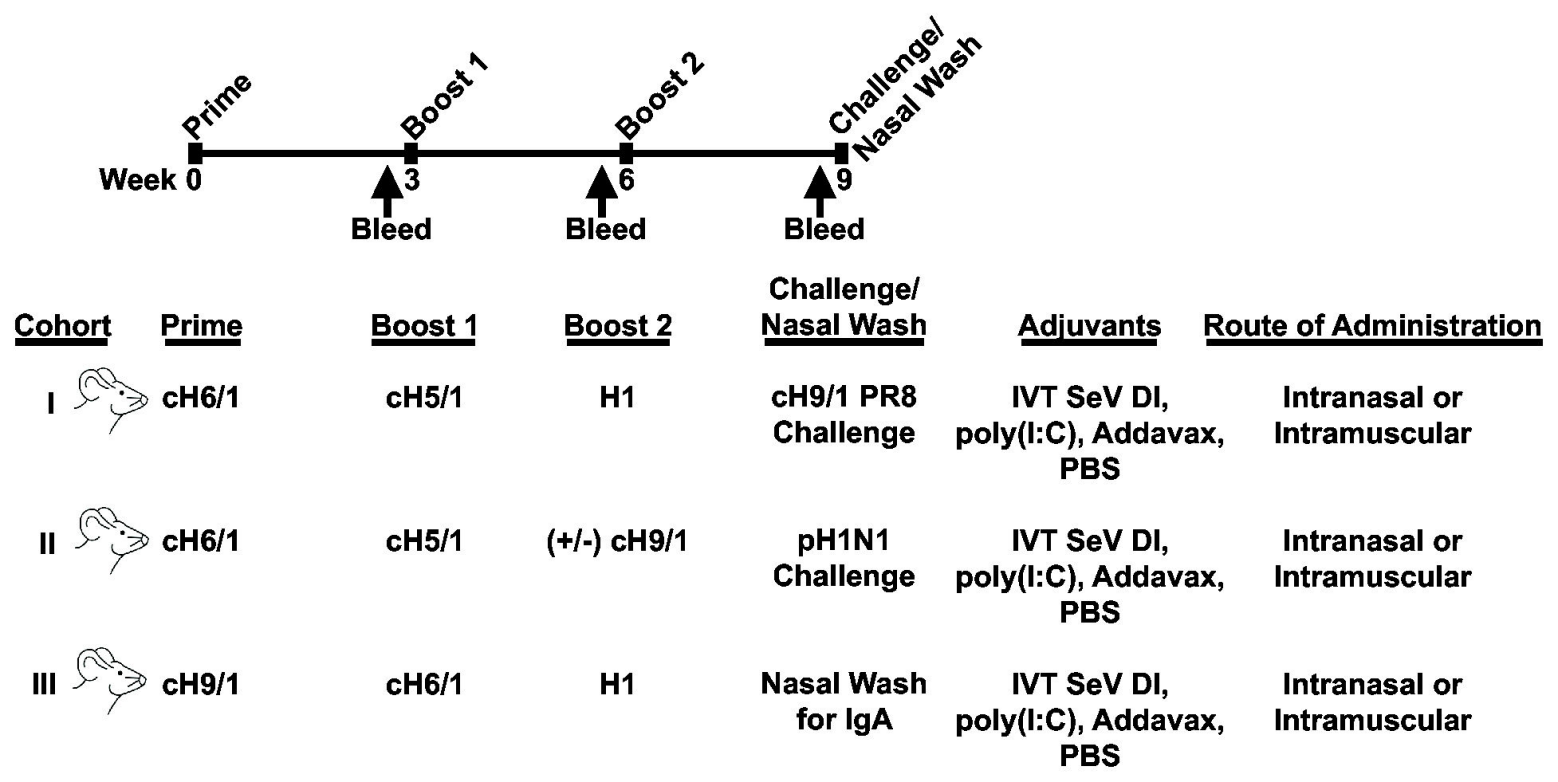
Immunization strategy and schedule. Three cohorts of mice were tested in this study with a variety of antigens and adjuvants. Different combinations of chimeric hemagglutinin proteins, all sharing a common stalk domain derived from influenza A/Puerto Rico/8/1934, were utilized such that each cohort remained naïve to various subtypes of hemagglutinin globular heads. Protein immunizations were delivered three weeks apart, mice were bled at the indicated time points, and challenge viruses were administered three weeks after the final immunization. In cohort II, half of the animals were challenged with pandemic H1N1 at week 6 and half were boosted with cH9/1 protein. In cohort III, animals were sacrificed and nasal washed for IgA in lieu of being challenged.

Naïve BALB/c mice 6-8 weeks of age received an initial protein immunization (prime) followed by boosts at three and six weeks after the prime. All vaccines were administered in a volume of 0.05 mL either intramuscularly (IM) or intranasally (IN). 10 µg of protein in PBS were administered for each immunization. IVT SeV DI and poly(I:C) were administered at 2 µg per dose, and Addavax was mixed with the antigen in PBS at a 1:1 ratio. Each cohort received the same adjuvant throughout the prime-boost-boost regimen. IVT SeV DI RNA was *in vitro* transcribed as previously described [12], and high molecular weight poly(I:C) and Addavax were purchased from Invivogen. Between 5-10 animals were used for each vaccine group. Cohort I: Each vaccine group received a regimen of soluble cH6/1, cH5/1 and H1 protein either intramuscularly or intranasally with an appropriate adjuvant: IVT SeV DI, poly(I:C) or Addavax (IM only). Cohort II: Ten animals for each vaccine group received a cH6/1 prime followed by a cH5/1 boost with an appropriate adjuvant. Half of these animals were challenged after two immunizations while the other half were boosted with cH9/1 protein. Cohort III: Sequential immunizations included cH9/1, cH6/1 and H1 protein. Control groups for each cohort included mice receiving unadjuvanted IM or IN administered cHA proteins, mice receiving poly(I:C) IN without protein antigen to control for the antiviral effect of adjuvants applied to the mucosa, and naïve animals (Figure 1). Notably, we have reported that the antiviral effects of IVT SeV DI RNA and poly(I:C) administered to the respiratory mucosa wane within ten days [12], but poly(I:C) administered intranasally in the absence of vaccine was included as a control nevertheless. Our group has previously evaluated the administration of poly(I:C) + BSA IM, and no antiviral effect was observed upon influenza virus challenge [9]. Other studies have demonstrated that MF59-like emulsions administered IM have no anti-influenza virus effect in the absence of an antigen [10]. The immunization strategies were devised such that cohort I remained naïve to the H9 subtype head, cohort II was naïve to the H1 subtype head, and cohort III was naïve to H2, H3 and H5 subtype globular heads. The order in which the immunogens are administered is apparently not important. For example, cH5/1 is interchangeable for cH6/1, cH9/1 or H1 proteins in terms of boosting stalk-directed antibodies and, therefore, kinetics of the developing immune response should not be affected by the order in which immunogens are administered. Also, previously published work from our group utilized a vaccination scheme where protein immunogens were administered in a different order and the regimen was equally effective [9]. 

### Viral challenge

Each influenza virus challenge was the equivalent of 10 murine median lethal doses (mLD50) and was administered IN in 0.05 mL of PBS to anesthetized animals 3 weeks after the final immunization. The cH9/1 virus (‘7+1’ reassortant in PR8) has a mLD50 of 10^3.67^ pfu and the mouse adapted pandemic H1N1 virus (A/Netherlands/602/2009) has a mLD50 of 5 pfu. Murine LD50 values were determined in 6-8 week old BALB/c mice [9]. Because mice are challenged after several months of immunizations, they are heavier and 10 LD50s may not induce the same percentage of body weight loss or 100% mortality. Animals were monitored daily for weight loss and humanely euthanized in accordance with institutional guidelines if they fell below 75% of their initial body weight. Statistical significance for survival curves was calculated by the Mantel-Cox test in Prism v6.0b (GraphPad Software). Statistical significance for weight loss curves was calculated using multiple unpaired t-tests correcting for multiple comparisons with the Holm-Sidak method in Prism v6.0b (GraphPad Software).

### Serum analysis and enzyme linked immunosorbant assay (ELISA)

 All ELISAs were performed using virus grown in 8 - 10 day old embryonated eggs. The allantoic fluid was clarified by centrifugation before being pelleted through a 30% sucrose cushion at 24,000 rpm with a SW-28 rotor (Beckman Coulter). Virus was resuspended in PBS and plated at 5 µg/mL. Stalk-specific HA titers were assayed by utilizing viral antigens with HA subtype globular heads to which the experimental animals were naïve [8]. Animals were bled 3 weeks after each immunization and sera were purified via centrifugation. Total IgG was assayed with a sheep anti-mouse IgG secondary antibody conjugated to HRP (GE Healthcare) and SigmaFast OPD substrate. Goat anti-mouse subtype and isotype specific secondary antibodies (Sigma) were detected with a rabbit anti-goat antibody conjugated to AP (Southern Biotech) and PNPP substrate (Invitrogen). Nasal washes for IgA antibodies were performed by flushing the upper respiratory tract with 0.5 mL of PBS. Statistical significance for ELISA curves was calculated using multiple unpaired t-tests correcting for multiple comparisons with the Holm-Sidak method in Prism v6.0b (GraphPad Software).

## Results

### Sequential chimeric HA protein immunizations induce a stalk-specific humoral response

 Three adjuvants were tested for their ability to boost HA stalk antibody titers. We have previously reported that IVT SeV DI RNA (RIG-I agonist) is an effective influenza virus vaccine adjuvant in the context of a homologous vaccine and challenge virus. In addition, poly(I:C) (MDA5/TLR3 agonist) and Addavax, which is sold commercially as an MF59 equivalent, have been reported by our group and others to be effective influenza virus vaccine adjuvants [12,15]. Here we describe the adjuvants’ effectiveness in boosting a broadly protective stalk response.

**Figure 2:**
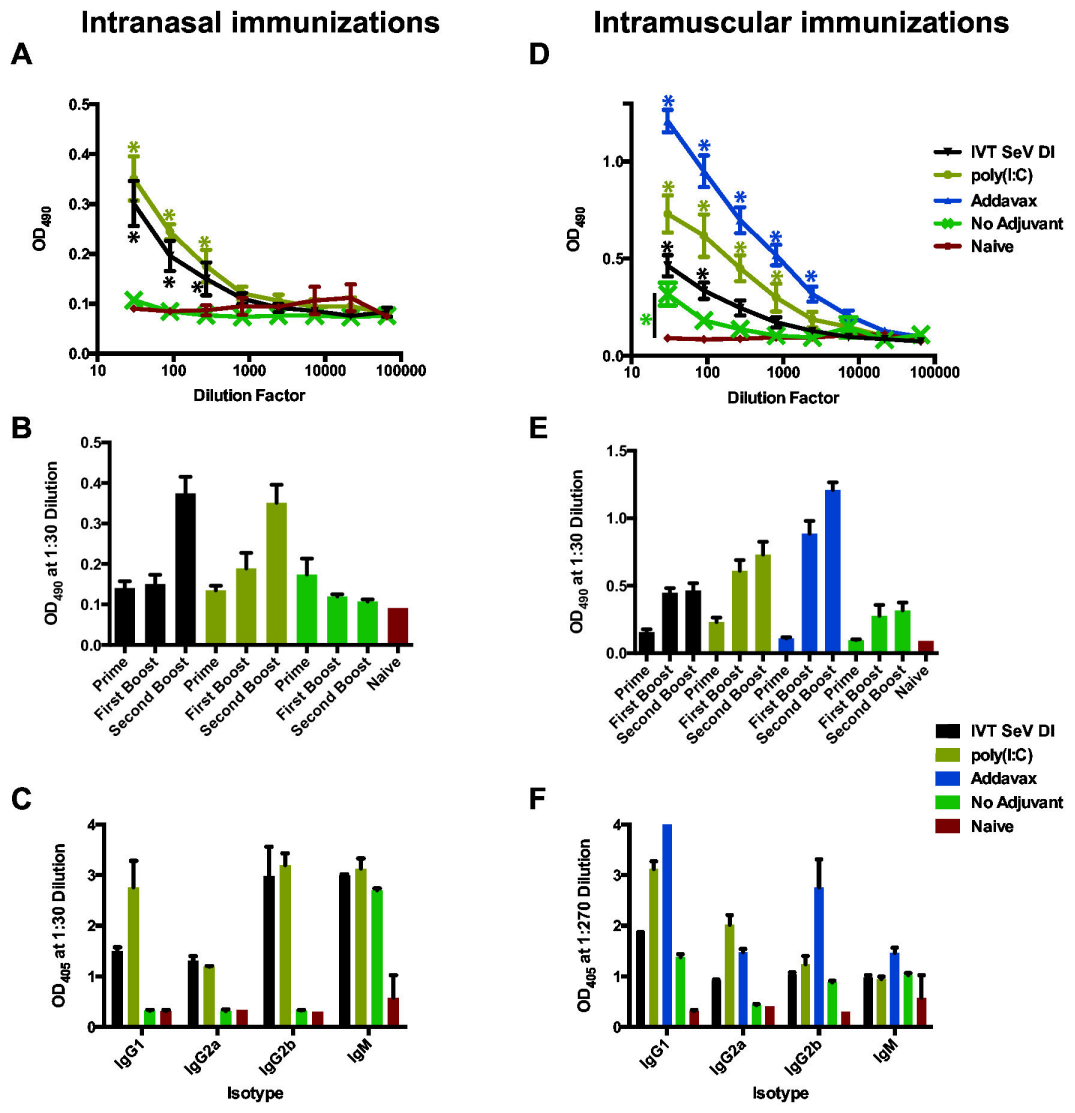
Sequential intranasal or intramuscular vaccination with chimeric hemagglutinins induces stalk reactive antibodies. ELISA was used to determine the HA stalk-specific seroreactivity induced by the vaccination strategy for cohort I (cH6/1, cH5/1 and H1 protein) to cH9/1 hemagglutinin in a 7+1 reassortant PR8 virus. Total stalk-directed IgG titers were assayed for sera after the third immunization for individual IN (A) or IM (D) vaccinees. *p<0.01. Significant differences in stalk-specific antibody titers were detected at the indicated points in the groups receiving adjuvant compared to the unadjuvanted control group (A & D). The unadjuvanted IM vaccinees had significantly higher titers compared to naïve mice (D). The HA stalk seroreactivity induced by each immunization demonstrates the effect of the protein prime-boost-boost strategy adjuvanted with IVT SeV DI, poly(I:C), Addavax or protein administered alone for the IN (B) and IM (E) routes of administration. Stalk-specific IgG antibodies were subtyped by ELISA for the IN (C) and IM (F) groups. (F) The IgG1 signal in the Addavax group was saturated at OD_405_=4. Error bars represent the standard error of the mean for each of five individual mice (A, B, D, & E) or the standard deviation for replicates of pooled serum samples (C & F). (C & F) Statistical analyses have not been included because replicates of pooled serum samples were assayed rather than biological replicates. The analyses for IN and IM immunizations in this figure were conducted independently and are not directly comparable.

 Cohort I consisted of several experimental groups that received sequential immunizations of soluble, trimerized cH6/1, cH5/1 and H1 protein (Figure 1). Immunization strategy I leaves animals naïve and hemagglutination inhibition (HI) negative to the subtype H9 head [9]. Animals were bled three weeks after each immunization, and HA stalk-specific serum titers were assayed by ELISA to the cH9/1 antigen (Figure 2). Stalk titers after three immunizations for the IN and IM groups are depicted in Figure 2, panels A and D, respectively. IVT SeV DI and poly(I:C) were effective mucosal adjuvants, while no stalk response was observed in response to cHA protein alone administered IN (Figure 2A). Unadjuvanted IM immunization induced a measurable stalk response which was significantly boosted by IVT SeV DI, Addavax and poly(I:C) (Figure 2D). Figures 2B & E depict the kinetics of the developing seroresponse. The IN route required three immunizations and the presence of an adjuvant to induce a strong stalk response (Figure 2B), while IM administration induced a measurable response after two adjuvanted immunizations (Figure 2E). In all cases, a single protein immunization did not generate a measurable stalk-directed humoral response (data not shown); however, it does apparently prime immunity and the response to subsequent antigen exposure.

 Adjuvants are known to direct the type of immune response from a given antigen depending upon their mechanism of action[16]. A higher ratio of IgG2a:IgG1 antibody subtypes is suggestive of a TH1 vs. TH2 type response. IVT SeV DI RNA and poly(I:C) both induced a range of IgG subtype antibodies in addition to IgM when administered IN (Figure 2C). Interestingly, the unadjuvanted protein given IN induced a substantial IgM response but no IgG response, suggesting that the presence of an effective mucosal adjuvant may be necessary for antibody class switching (Figure 2C). Each of the adjuvanted IM immunizations induced a variety of IgG subtypes (Figure 2F).

### Sequential cHA protein immunizations induce HA stalk-mediated protection to influenza virus challenge

**Figure 3:**
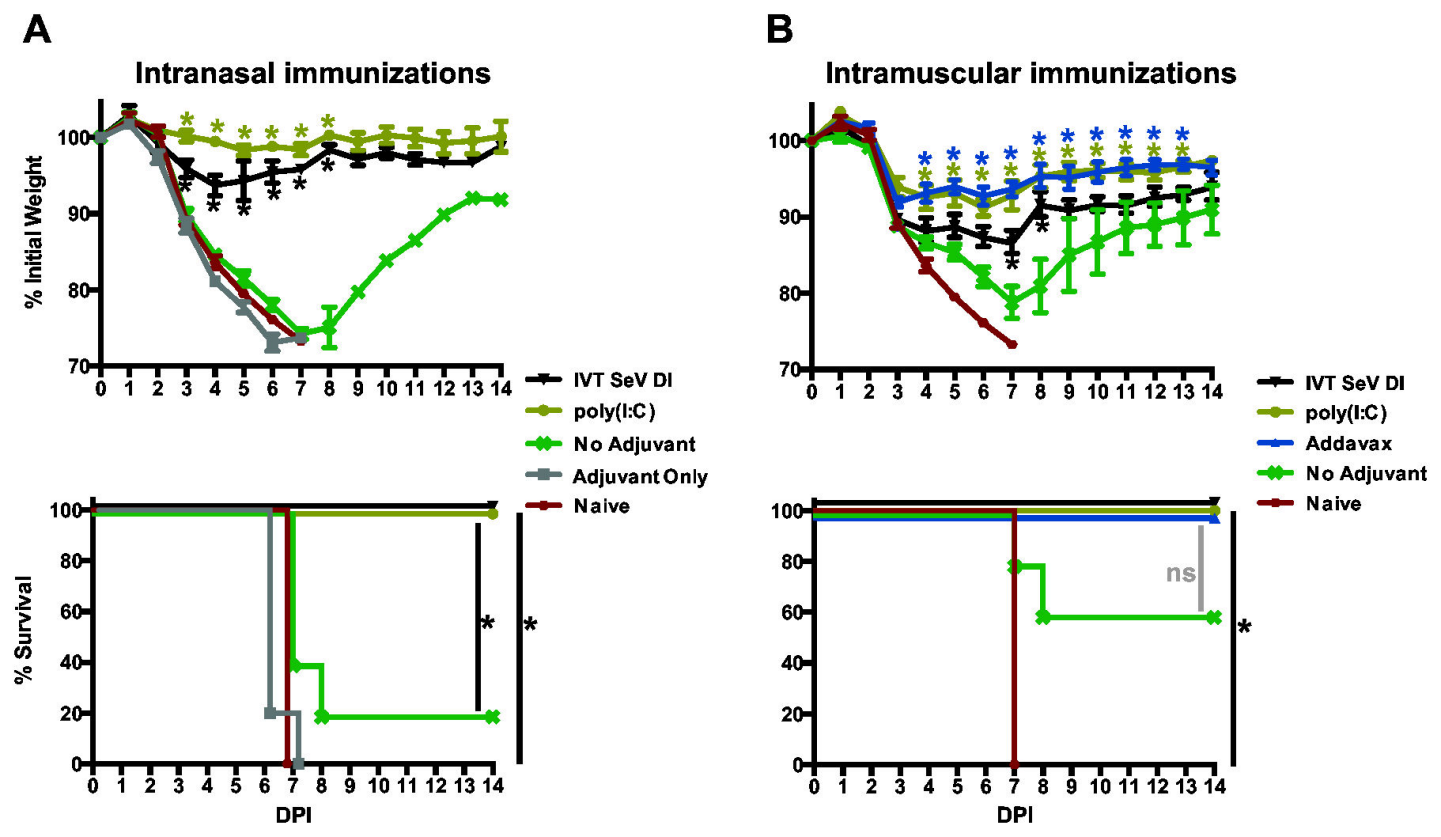
Stalk-directed immunization strategies are protective using intranasal or intramuscular routes of administration. (A) Three sequential intranasal vaccinations (cohort I: cH6/1, cH5/1 and H1 protein) adjuvanted by either IVT SeV DI or poly(I:C) serve to protect mice against infection with 10 mLD50 of the cH9/1 PR8 ‘7+1’ reassortant. Administration of poly(I:C) alone (Adjuvant Only) did not have an antiviral effect. *p<0.0001. Significant differences in weight loss for IN vaccinees were observed at the indicated points compared to the unadjuvanted and the adjuvant only groups (A, top panel). (B) The same vaccination regimen administered intramuscularly was also highly efficacious at preventing weight loss and mortality when adjuvanted with IVT SeV DI, poly(I:C) and Addavax, while partial protection for mortality and weight loss was observed for the unadjuvanted vaccine group compared to naïve controls. *p<0.01. Significant differences in weight loss in the IM vaccinees were observed at the indicated points compared to the unadjuvanted group (B, top panel). (A & B)The curves for the naïve animals depict the same data for the weight loss and survival curves. (A & B, bottom panels) Statistical significance for Kaplan-Meier survival curves was calculated with the Mantel-Cox test (* p<0.02). The difference in survival between the adjuvanted and unadjuvanted IM vaccinated groups were not significant (ns) at this viral challenge dose.

 The animals from cohort I (Figure 1) were subsequently challenged with 10 mLD50 of a ‘7+1’ reassortant of the PR8 influenza virus strain expressing cH9/1 HA. Therefore the stalk of the challenge virus is homologous to the cHA immunogens, but the mice are naïve to the subtype H9 globular head. Protection from the challenge was associated with stalk-directed antibody titers (Figure 2), and IVT SeV DI and poly(I:C) adjuvanted IN immunizations significantly minimized morbidity as measured by weight loss (Figure 3A, top panel) and protected against mortality (Figure 3A, bottom panel). IVT SeV DI, poly(I:C) and Addavax were all effective at significantly minimizing weight loss and preventing mortality in the IM immunized animals (Figure 3B). Despite the higher titers induced by Addavax in comparison to IVT SeV DI RNA and poly(I:C) (Figure 2D), the animals lost weight at similar rates (Figure 3B). In contrast to unadjuvanted IN vaccinations, unadjuvanted IM vaccination improved survival compared to naïve animals. In summary, we show that adjuvants improve the efficacy of cHA stalk-directed vaccine constructs.

### Adjuvanted cHA immunizations induce a broadly reactive seroresponse to a panel of H1 subtype influenza A viruses

**Figure 4:**
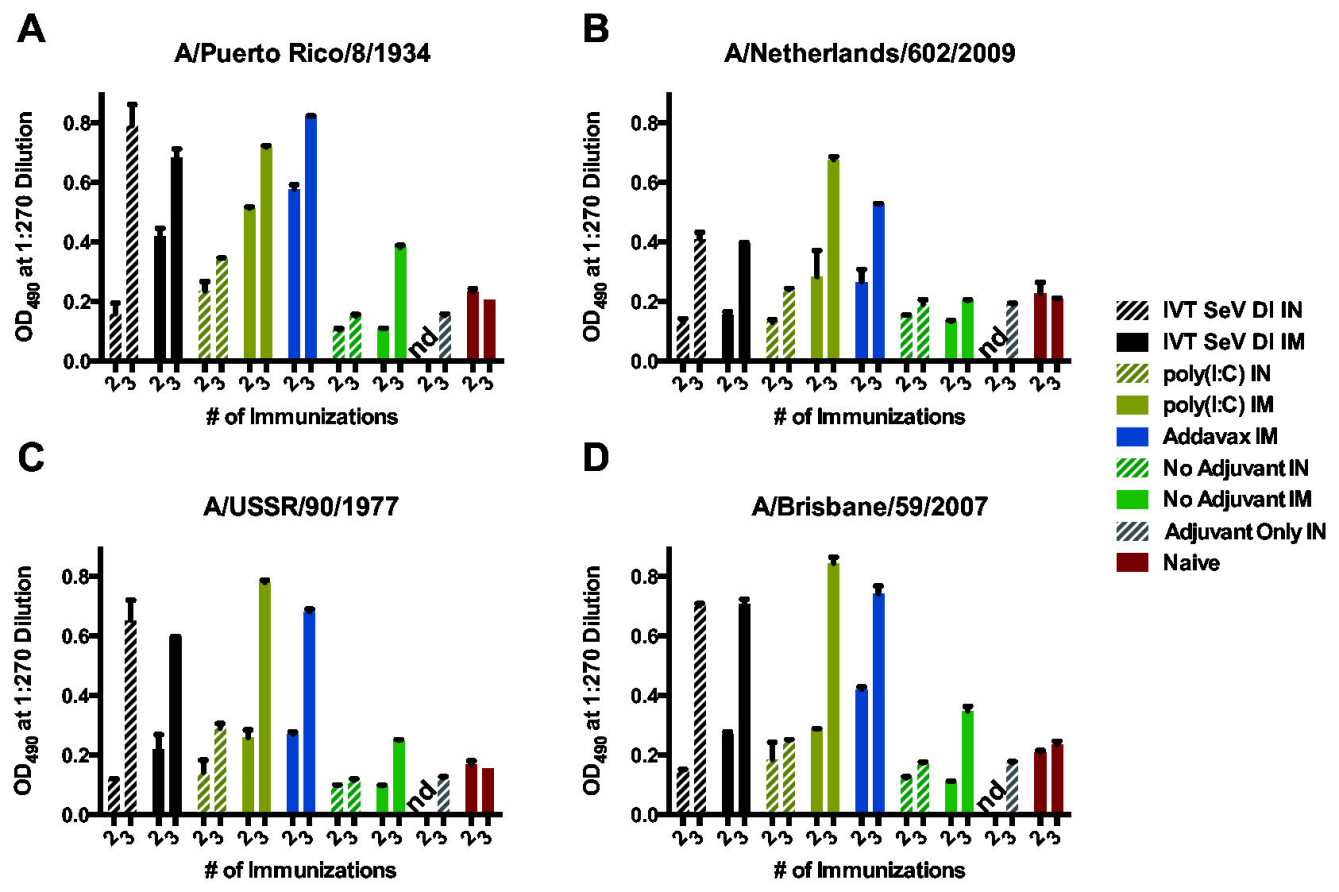
Stalk-specific seroreactivity to subtype H1 influenza viruses induced by cHA immunizations. ELISA depicts the seroreactivity of twice and thrice intranasally or intramuscularly vaccinated mice (cohort II: cH6/1, cH5/1, +/- cH9/1) to a variety of subtype H1 influenza viruses including (A) A/Puerto Rico/8/34 which has a stalk domain homologous to the cHA immunogens. Seroreactivity to heterologous H1N1 subtype strains including (B) pH1N1 (A/Netherlands/602/2009) and two drifted seasonal isolates, (C) A/USSR/90/1977 and (D) A/Brisbane/59/2007, is also demonstrated by ELISA. Error bars represent the standard deviation for replicates of pooled serum samples. Animals receiving two administrations of poly(I:C) alone (Adjuvant Only) IN were not bled at this timepoint so no data (nd) were generated.

 In order to determine if our adjuvanted immunization strategies induce a broad response to a range of drifted seasonal and pandemic H1 viruses, animals in cohort II were immunized with cHA proteins such that they remained naïve to the subtype H1 globular head (Figure 1). Serum samples from animals in each group of cohort II were pooled and assayed for reactivity to a range of subtype H1 viruses covering more than 80 years of drift from the PR8 stalk immunogen (Figure 4). We observed broad seroreactivity after both IM or IN immunization with adjuvanted protein. In Figure 4A, stalk reactivity is confirmed for the homologous stalk antigen and seroreactivity is demonstrated for all H1 stalks tested including the pandemic 2009 H1N1 virus (Figure 4B-D). Inclusion of an adjuvant boosted cross reactive antibody titers compared to soluble HA immunization alone. We note experimental variability between the observed stalk-specific seroreactivity for animals receiving IN immunizations adjuvanted with poly(I:C) in cohorts I and II relative to mice receiving IVT SeV DI intranasally (Figures 2 and 4). Previous reports show that monoclonal stalk antibodies and polyclonal sera directed at the HA stalk may be effective *in vivo* when passively transferred or in an *in vitro* neutralization assay [9,14]. Virus neutralization by trypsin-inactivated sera was not observed at the limit of detection of a microneutralization assay (serum dilution of >1:160; data not shown). In addition to virus neutralization, other mechanisms of action for broadly reactive antibodies including antibody-dependent cellular cytotoxicity [17] and complement dependent lysis [18] have also been described and may have *in vivo* relevance.

### Adjuvanted cHA immunizations are protective against heterologous challenge with the 2009 pandemic H1N1 virus

**Figure 5:**
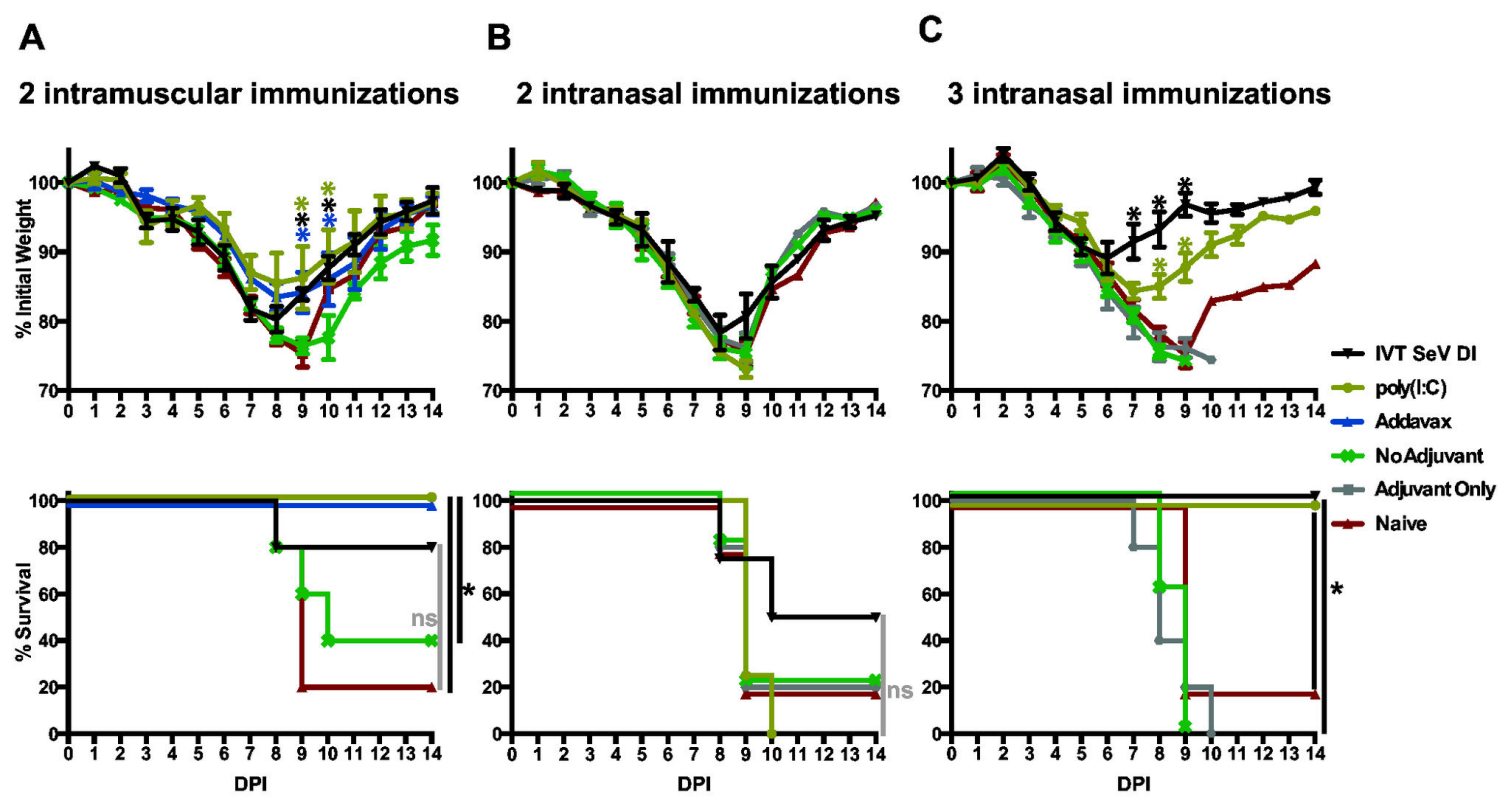
Two adjuvanted IM immunizations induce protective, stalk-mediated immunity while three are necessary for IN administration. (A) Two IM immunizations (cohort II: cH6/1 and cH5/1 protein) adjuvanted with IVT SeV DI, poly(I:C) or Addavax were sufficient to limit morbidity measured by weight loss induced by 10 mLD50 of mouse adapted pH1N1 virus. (A, top panel) *p<0.005. Significant differences in weight loss were observed at days 9 and 10 for each of the adjuvanted groups when compared to the unadjuvanted control. (A, bottom panel) Addavax and poly(I:C) resulted in a significant reduction in mortality to challenge compared to the naïve (*p<0.02) and unadjuvanted (*p<0.05) animals, while other survival differences are not statistically significant (ns). (B) Two IN immunizations (cohort II: cH6/1 and cH5/1 protein) were insufficient to protect mice from a 10 mLD50 challenge with mouse adapted pH1N1 virus. (C) An additional five mice in cohort II were immunized a third time with cH9/1 protein before being challenged with pH1N1. (C, top panel) *p<0.0001. Significant differences in weight loss were observed at the indicated points compared to the unadjuvanted control group. (C, bottom panel) The animals receiving adjuvanted vaccine had a significant improvement in survival compared to unadjuvanted and naïve animals (*p<0.02). (A & B) The curves for the naïve animals depict the same data set for weight loss and survival. (A & C, bottom panels) Statistical significance for Kaplan-Meier survival curves was calculated with the Mantel-Cox test. The Adjuvant Only group is poly(I:C) administered IN in the absence of antigen.

In order to determine if two adjuvanted cHA protein immunizations are sufficient to provide mice with protection to a heterologous viral challenge, half of the animals in cohort II were challenged with 10 mLD50 of the 2009 pandemic H1N1 virus (A/Netherlands/602/2009, mouse adapted) after two immunizations. The other half of cohort II received a third immunization with cH9/1 protein. In Figure 5A, weight loss curves and Kaplan-Meier survival curves indicate that two cHA immunizations adjuvanted with IVT SeV DI, poly(I:C) or Addavax are sufficient to reduce weight loss and protect animals from a lethal infection. This protection is based upon immunity to a heterologous (drifted) HA stalk. One hundred percent survival and significant reduction in weight loss was observed in the poly(I:C) and Addavax groups, while the IVT SeV DI improved survival to 80% compared to 40% for the unadjuvanted group and 20% for the naïve animals. Animals receiving two intranasal vaccinations were not protected from infection with 10 mLD50 of the pH1N1 virus (Figure 5B), as would be expected based upon their relatively low serum antibody titers to the challenge virus (Figure 4B). However, a third IN immunization significantly boosted stalk reactive antibodies in the sera (Figure 4B) and protected the animals in the IVT SeV DI and poly(I:C) groups from weight loss and mortality when challenged with the heterologous pH1N1 virus (Figure 5C).

### Adjuvanted cHA protein immunizations induce heterosubtypic stalk reactivity

**Figure 6:**
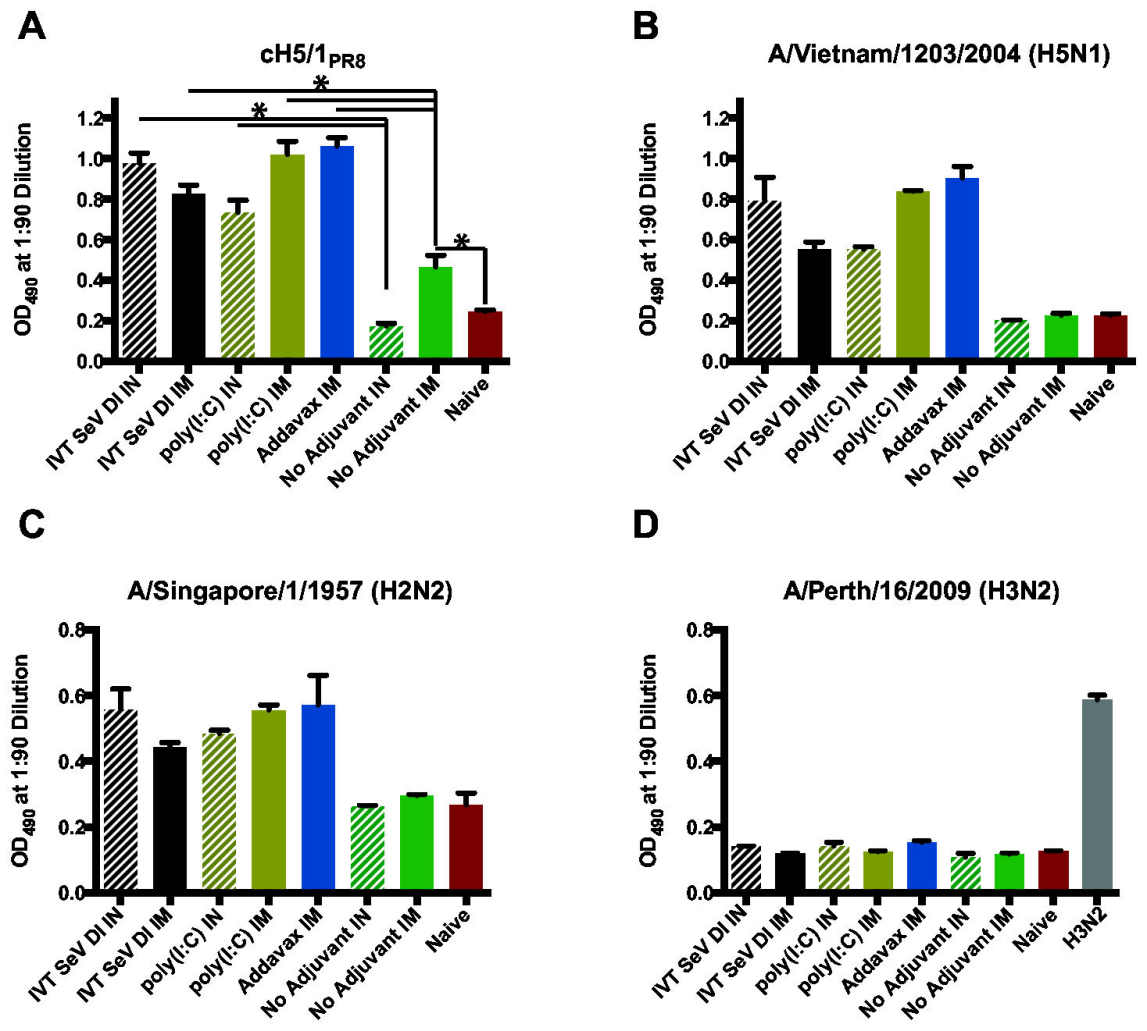
Adjuvanted H1 stalk immunization induces seroreactivity to heterosubtypic group I but not group II hemagglutinins. Mice sequentially immunized (cohort III: cH9/1, cH6/1 and H1 soluble protein) are seroreactive to the homologous PR8 stalk (A), the heterosubtypic H5 (B) and H2 subtype stalks (C), but not to the group II subtype H3N2 virus (D). The H3N2 positive control is sera from mice infected with a sublethal dose of X31 (A/Hong Kong/1968 HA and NA in the PR8 background). Error bars represent the standard error of the mean for individual mice (A) or the standard deviation for replicates of pooled serum samples (B-D). *p<0.001. Significant differences in stalk-specific HA antibody titers were observed at the indicated points compared to the unadjuvanted control group.

 In order to further define the breadth of reactivity stimulated by adjuvanted cHA immunizations, a third cohort (Figure 1) was immunized such that mice remained naïve to the subtypes H5 and H2 head domains. H5N1 influenza virus is considered a major threat for the next pandemic, and H2N2 influenza virus was responsible for the 1957 pandemic and could hypothetically reemerge in humans. Therefore, a broadly protective group I influenza virus vaccine should induce a seroresponse to H2 and H5 subtype HAs in addition to H1 subtype viruses. In Figure 6A, reactivity to the homologous HA stalk is confirmed using the cH5/1 virus. Seroreactivity for mice in cohort III was also observed to subtypes H5 (Figure 6B) and H2 HAs (Figure 6C); however, the seroreactivity was apparently limited to group I HAs as the antisera did not recognize subtype H3 HA from group II (Figure 6D).

### Intranasal vaccination with adjuvanted HA subunit vaccine induces HA stalk-specific IgA production

**Figure 7:**
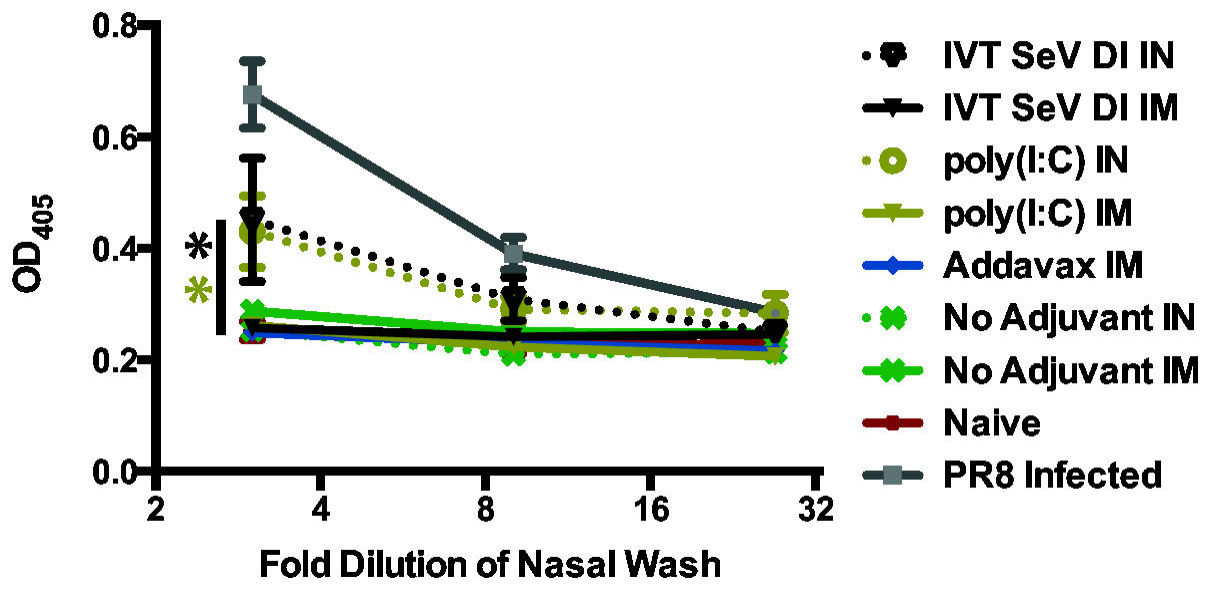
Intranasal immunization induces HA stalk-specific IgA antibodies. Mice from cohort III (cH9/1, cH6/1 and H1 soluble protein) that were vaccinated IN (dotted lines) with the addition of IVT SeV DI or poly(I:C) had detectable, HA stalk-specific IgA in the nasal washes. IM vaccinees (solid lines) did not have detectable stalk-specific IgA titers in nasal washes. Nasal washes of mice sublethally infected with influenza A/Puerto Rico/8/1934 were included as a positive control for reference. Error bars represent standard error of the mean for individual mice. * p<0.02. Significant differences in stalk-specific IgA titers in nasal washes were observed when comparing IN to IM immunization for both the IVT SeV DI and poly(I:C) groups.

 The animals from cohort III (Figure 1) were sacrificed and the upper respiratory tract was washed with PBS to assay for the presence of IgA. IgA is an important component of mucosal immunity and has been shown to play a role in preventing influenza virus infection [19–21]. Live attenuated vaccines have been recognized for the ability to induce mucosal IgA as well as serum IgG, while traditional inactivated vaccines administered IM primarily produce an IgG seroresponse. In Figure 7, we demonstrate that adjuvanted protein induced mucosal IgA production specific to the HA stalk when administered intranasally. IM vaccination did not induce measurable IgA titers to the HA stalk. These data indicate that IN administration of subunit or killed influenza virus vaccines may have advantages over IM immunization for its ability to induce IgA; however, further research is needed to establish a protective role for IgA in vaccinated laboratory animals and humans [22].

## Discussion

Analyses of human antisera have established that humans produce HA stalk antibodies after infection with influenza A virus and that associated memory B cells may be long lived [8,23,24]. The levels of circulating HA stalk antibodies may wane after infection until falling below protective levels such that a person may be infected again. The next generation of influenza vaccines must provide broad and persistent humoral immunity similar to what is observed soon after a viral infection. The most commonly administered seasonal vaccines seem not to substantially boost this type of antibody response [25]. We suggest that an effective vaccine formulation may consist of a rationally designed antigen combined with a safe, effective adjuvant that induces broad seroreactivity to the influenza hemagglutinin stalk. 

We have previously reported that influenza virus infection induces high stalk-specific antibody titers in humans [8]. This phenomenon can be modeled in mice which can be subsequently boosted with protein immunizations to yield protective, stalk-directed immunity [9]. While replicating viruses effectively induce a cross-reactive humoral response, the development of formulations that may be safe and effective in naïve individuals (e.g. children) is an important component in the development of a universal influenza vaccine. Here, we demonstrate that HA protein may be effective when administered mucosally or intramuscularly with a variety of adjuvants including the IVT SeV DI RNA. Vaccination with the subtype H1 stalk is sufficient to induce broad reactivity to diverse group I HA stalks, including subtypes H1, H2 and H5, on the basis of highly conserved epitopes.

The use of a RIG-I agonist, IVT SeV DI RNA, effectively boosted broadly-reactive HA stalk antibodies and induced protective immunity comparable to well established adjuvants including poly(I:C) and Addavax. Our results indicate that HA subunit vaccines may also enhance HA stalk-directed mucosal immunity in combination with an adjuvant, including IVT SeV DI, when administered IN. IgA may play an important role in preventing influenza virus transmission [19–21], and HA stalk antibodies were only detectable with IN vaccination. Either route of administration is sufficient to induce a robust HA stalk IgG response and establishes that an effective adjuvant may be an important component in the development of stalk-directed universal influenza vaccines. While a wide variety of adjuvants are under development, safety concerns represent a major barrier for clinical implementation. Our data indicate that many adjuvants and routes of administration are effective for inducing broad immunity to influenza virus, which promises researchers and physicians sufficient flexibility to address safety issues and optimize the nature of immunity induced as new vaccine formulations are developed.
